# Addition of α-*O*-GlcNAc to threonine residues define the post-translational modification of mucin-like molecules in *Trypanosoma cruzi*

**DOI:** 10.1007/s10719-013-9469-7

**Published:** 2013-02-21

**Authors:** Lucia Mendonça-Previato, Luciana Penha, Tatiana Cortes Garcez, Christopher Jones, Jose Osvaldo Previato

**Affiliations:** 1 Laboratório de Glicobiologia, Instituto de Biofísica Carlos Chagas Filho, Universidade Federal do Rio de Janeiro, 21 941 902 Ilha do Fundão, Cidade Universitária, Rio de Janeiro, RJ Brazil; 2 National Institute for Biological Standard and Control, Potters Bar, Hertfordshire, EN6 3QG UK

**Keywords:** *Trypanosoma cruzi*, Posttranslational modification, Mucins, pp-α-GlcNAcT, *trans*-sialidase

## Abstract

*Trypanosoma cruzi*, an intracellular protozoan etiologic agent of Chagas disease is covered by a dense coat of mucin-type glycoproteins, which is important to promote the parasite entry and persistence in the mammalian host cells. The *O*-glycosylation of *T*. *cruzi* mucins (Tc-mucins) is initiated by enzymatic addition of α-*O*-*N*-acetylglucosamine (GlcNAc) to threonine (Thr) by the UDP-GlcNAc:polypeptide α-*N*-acetylglucosaminyltransferase (pp-α-GlcNAcT) in the Golgi. The Tc-mucin is characterized by the presence of a high structural diversity of *O*-linked oligosaccharides found among different parasite strains, comprising two *O*-glycan Cores. In the Core 1, from strains principally associated with the domestic transmission cycle of Chagas disease, the GlcNAc *O*-4 is substituted with a β-galactopyranose (βGal*p*) unit, and in the most complex oligosaccharides the GlcNAc *O*-6 is further processed by the addition of β1 → 2-linked Gal*p* residues creating a short linear Gal*p*-containing chain. In the Core 2 structures, expressed by strains isolated from *T*. *cruzi* sylvatic hosts, the GlcNAc *O*-4 carries a β-galactofuranose (βGal*f*) unit and the GlcNAc *O*-6 can carry a branched Gal*p*β1 → 3[Gal*p*β1 → 2]Gal*p*β1 → 6 motif. The *O*-glycans carrying nonreducing terminal βGal*p* are available for sialylation by a surface *T*. *cruzi trans*-sialidase activity. Based on structural results, this review summarizes available data on the highly conserved process, which adds the GlcNAc unit in α-linkage to Thr residues the basis of the post-translational modification system in *T*. *cruzi* mucins. In addition, a mechanism unique employed by the parasite to transfer exogenous sialic acid residues to Tc-mucins is presented.

## Introduction

Chagas’ disease, an infection caused by the protozoan *Trypanosoma cruzi*, remains a major cause of morbidity in Latin America. Though major advances in preventing the spread of this disease have been made in recent decades, an estimated 10 million people are infected due to prior exposure to *T*. *cruzi* [1], and about 30 % of the individuals infected are characterized by heart inflammation and dysfunction [2]. *T*. *cruzi* presents genetic diversity, resulting in the prevalence of specific clinical forms and morbidity of Chagas disease, partially due to different protein expression levels and genomic instability [3]. Zingales and co-authors [4] subdivided *T*. *cruzi* species into six Discrete Typing Units (DTUs) designated *T*. *cruzi* I to *T*. *cruzi* VI. Recently, *T*. *cruzi* I has been correlated with cardiomyopathy manifestations [5, 6] increasing the need for further comparative biological and biochemical studies on different *T*. *cruzi* strains.

Protein glycosylation is an important post-translational modification underlying host-parasite interactions, which may determine the outcome of infection. The surface of *T*. *cruzi* is covered principally by a family of sialylglycoproteins (*T*. *cruzi* mucins) linked to the cell membrane through a glycosylphosphatidylinositol (GPI) anchor [7]. The protein domain is rich in threonine residues [8, 9] which can be modified with multiple *O*-linked glycan chains [10]. These *O*-glycans are acceptors of sialic acid derived from exogenous sialylglycoconjugates, through a reaction catalysed by a trypanosomal-specific *trans*-sialidase [11, 12]. The post-translational modifications of *T*. *cruzi* mucins (Tc-mucin) give rise to *O*-linked glycans attached to the peptide by α-GlcNAc-*O*-Thr linkages [13], through the activity of a unique UDP-GlcNAc:polypeptide α-*N*-acetylglucosaminyltransferase (pp-α-GlcNAcT) [13]. In contrast, in the mammalian mucins *O*-glycosylation, *N*-acetylgalactosamine (GalNAc) units are attached through α-glycosidic linkage to the Ser and Thr residues [14–16].

The core α-GlcNAc-*O*-Thr of Tc-mucins is further processed by β-galactopyranose (βGal*p*) (Core 1) and β-galactofuranose (βGal*f*) (Core 2) units in a *T*. *cruzi* strain-specific pattern of linkages and substitutions [10, 17]. Here we highlight the *T*. *cruzi* UDP-GlcNAc:polypeptide α-*N*-acetylglucosaminyltransferase (pp-α-GlcNAcT) and *trans*-sialidase activities, and unique *O*-glycan assemblies in Tc-mucins. Proven functions for the glycan domains of Tc-mucins on the pathogenesis of Chagas disease are unknown, although potential functions are addressed in this review. In fact, different strains of *T*. *cruzi* form a very heterogeneous group with specific characteristics such as histotropism, antigenicity, infectivity and pathogenicity [18], suggesting that the interaction of the parasite and human host cells would determine the severity of Chagas’ disease. However, so far, the direct correlation of the structure of Tc-mucins O-glycans and the immunopathology of the disease has not been characterized.

## *T*. *cruzi* UDP-GlcNAc:polypeptide α-*N*-acetylglucosaminyltransferase (pp-α-GlcNAcT)

**Fig. 1:**
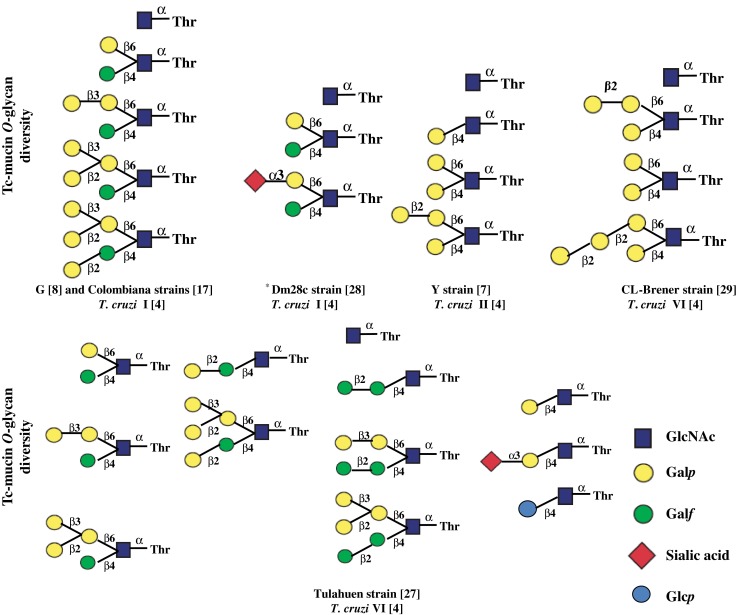
The structures of the derived glycan of -O-Thr Tc-mucins from different *T*. *cruzi* strains. Representative glycan structure is indicative with *colored geometric symbols*, conform to those recommended by Consortium for Functional Glycomics

The post-translational modification of Tc-mucin with *O*-linked 2-*N*-acetamido-2-deoxy-D-glucopyranose (*O*-GlcNAc) is conserved in all *T*. *cruzi* strains studied to date (Fig. 1). Direct compositional analyses of Tc-mucin core proteins show that Thr are much more frequent than Ser residues [8, 9]. The same fact occurs in *T*. *cruzi* MUC gene-derived protein sequences [9] (Table 1). The α-anomeric configuration of the protein-*O*-linked GlcNAc was determined by 2D-Nuclear Magnetic Resonance Spectroscopy (NMR) analysis of Smith-degraded sialylglycoproteins [13]. The key data were the ^3^ *J*_H1,H2_ coupling constant, which is small, and ^1^ *J*_H1,C1_, determined in an HSQC spectrum without ^13^C decoupling. Both these techniques, combined with the chemical shift data and the resistance of the product of *in vitro* enzymatic GlcNAc addition to a synthetic peptide substrate (KP_2_T_8_KP_2_) to digestion with jack bean β-*N*-acetylglucosaminidase, indicate that the GlcNAc residue has the α-anomeric configuration, thus distinguishing this system from the single β-linked GlcNAc residue observed as dynamic glycosylation on a number of nuclear and cytosolic proteins, and which is believed to serve a regulatory purpose [19, 20]. The kinetic properties of the *T*. *cruzi* pp-α-GlcNAcT were investigated using microsomal fractions prepared from insect-dwelling (epimastigotes) and cell-derived trypomastigote forms of *T*. *cruzi II* (DTU) [4] Y-strain, [7], the synthetic peptide acceptor KPPTTTTTTTTKPP, and with UDP-[^3^H]GlcNAc as the sugar donor [13, 21]. The enzyme has an optimal pH of 7.5 to 8.5 and requires the presence of Mn^2+^. It is strongly inhibited by UDP but unaffected by the presence of tunicamycin or amphomycin, indicating that activated dolichol donor intermediates are not involved [13]. The microsome system from *T*. *cruzi* is unable to add [^3^H]GlcNAc to the synthetic nonapeptide YSDSPSTST [22], the substrate for *O*-linked β-*N*-acetylglucosamine transferase (OGT) an enzyme which catalyses a common post-translational modification of nuclear and cytoplasmic proteins [19, 20].

**Table 1:** Amino acid composition of Tc-mucins purified from different *T*. *cruzi* strains

^a^Amino acid	*T*. *cruzi* strain				
	G	Y	CL-Brener	Tulahuen	^b^MUC1-Y [9]
Thr	50.3	45.9	53.8	50.5	54.5
Asp/Asn	12.8	11.2	10.0	12.9	4.5
Ala	10.3	10.1	8.7	10.6	5.7
Pro	9.3	8.4	7.8	6.3	11.4
Ser	0.9	1.1	0.9	0.9	4.6
Glu/Gln	5.2	6.9	5.4	5.7	5.7
Lys	0.6	4.4	0.6	2.1	3.4
Gly	7.1	8.5	6.8	7.8	0.0
Arg	0.0	0.4	0.3	0.0	4.6
Val	2.3	1.6	3.2	2.3	1.1
Met	0.0	0.0	0.0	0.0	0.0
Ile	0.4	0.7	0.8	0.3	3.4
Tyr	0.0	0.0	0.0	0.0	0.0
Leu	0.5	0.5	0.5	0.5	0.0
His	0.4	0.3	0.5	0.0	0.0
Phe	0.0	0.0	0.0	0.0	0.0

^a^Amino acid content is indicated in number of residues per 100 residues

^b^Composition deduced from the gene sequence

In *T*. *cruzi* a gene which encodes pp-α-GlcNAcT activity was identified, designed TcOGNT-2 [23], and the predicted sequence is 61–81 % similar to the 250-amino-acid catalytic domain of DdGnt2, a *Dictyostelium discoideum* membrane-bound Golgi pp-αGlcNAcT [24, 25]. Recently, it was demonstrated that TcOGNT-2 shows different levels of expression during the life cycle of *T*. *cruzi*. When trypomastigotes penetrate Vero cells and differentiate into amastigotes, TcOGnT-2 expression declines, and low levels of TcOGNT-2 mRNA and protein were detected. Later, when intracellular amastigotes differentiate in trypomastigotes the TcOGNT-2 increases expression again [26]. Interestingly, overexpressing of TcOGNT-2 enhances *T*. *cruzi* infectivity [26].

## The assembly of Thr-linked *O*-glycans of Tc-mucins

The assembly of Thr-linked *O*-glycans of Tc-mucins is initiated in the Golgi [13, 21] by the pp-α-GlcNAcT. The *O*-α-GlcNAc residues are further processed to form the backbone structure for biologically important epitopes. The addition of galactopyranose and galactofuranose monosacharides is catalyzed by β1,6; β1,4; β1,3; β1,2-Gal*p* transferases and β1,4; β1,2-Gal*f* transferases, and the attachment of sialic acid at some terminal βGal*p* residues is catalyzed by a *trans*-sialidase activity [7].

The simplest glycosylation pattern found in Tc-mucin consists of a single unsubstituted *O*-linked GlcNAc residue. In the *T*. *cruzi* Tulahuen strain [27], high performance liquid chromatography (HPLC) analyses suggest that single GlcNAc residues are present at about 20 % of the glycosylation sites, and similarly high amounts are present in the *O*-glycan mixture from other strains [7, 8, 17, 28, 29] (Fig. 1). *O*-glycans were isolated as alditols from purified Tc-mucins by alkaline reductive cleavage and fractionated by gel filtration chromatography [8]. The purification is difficult due to high levels of glycosylation with eventual sialylation, thus when it is required further purification by HPLC on pyrolysed graphitic carbon (PGC) was performed [27–29]. *O*-glycan chemical structures from Tc-mucins were determined, usually, by one- and two-dimensional (2D) homo- and hetero-nuclear NMR spectroscopy combined with methylation analysis, mild acid hydrolysis and mass spectrometry.

Two options for the addition of the first Gal residue have been identified. The Core 1, in the Y [7] and CL-Brener [29] strains, a βGal*p* residue is transferred to the GlcNAc *O*-4 (Fig. 1). The core 2, in the G [8], Colombiana [17], and Dm28c [28] strains, a βGal*f* residue is attached at GlcNAc *O*-4 to give structures shown in Fig. 1. The *O*-glycans from *T*. *cruzi* Tulahuen strain mucins show high structure diversity [27]. The Cores 1 and 2 are synthesized by Tulahuen strain, the Gal*p* β → 4GlcNAc (Core 1) and Gal*f*β → 4GlcNAc (Core 2). Surprisingly, sialylation is the only observed elaboration of the Gal*pβ* → 4GlcNAc [27] (Fig. 1). In the Tulahuen strain [27] besides both Gal*p*β → 4GlcNAcα → Thr and Gal*f* β → 4GlcNAcα → Thr there is also evidence for *O*-glycan with a β-glucose residue on GlcNAc *O*-4. This appears to be a “dead-end” species, as no higher oligosaccharides with this glucosylation pattern have been observed.

## Extension of the GlcNAcα1-/Thr

In the CL and Y *T*. *cruzi* strains, more complex glycan structures arise from the attachment of a β-Gal*p* residue at GlcNAc *O*-6, leading to a disubstituted oligosaccharide (Fig. 1). Further elaborations occur by addition of one or two β-Gal*p*(1 → 2) residues to the Gal*p* present on the GlcNAc *O*-6, thus a short linear galactan chain is created on the 6-arm [27, 29]. Significant in the Core 1 structure is the presence of *O*-glycans terminated by Gal*p*α1 → 3. In Tc-mucins isolated from cell-derived trypomastigotes the *O*-glycans contain the trisaccharide Gal*p*α1 → 3Gal*p*β → 4GlcNAcα → [30]. The α-galactosylated glycans are highly immunogenic to humans and represent the major epitope for trypanolytic anti-αGal antibodies found in the serum of acute and chronic chagasic patients [30, 31].

Three biosynthetic pathways by which Core 2 Gal*f*β → 4GlcNAcα → are elaborated and have been characterized [27]. The first is similar to that seen in the Core 1, with addition of a βGal*p* residue to the GlcNAc *O*-6, to give rise to disubstituted structure (Fig. 1). This has been observed for the G-, Colombiana-, Tulahuen- and Dm28c- strains [8, 17, 27, 28]. Second, in the Tulahuen strain, glycans resulting from the addition of either β-Gal*p*1 → 2 or β-Gal*f*β1 → 2 to the Gal*f*β → 4GlcNAcα → are observed, leading to approximately equal amounts [27]. Third, the expression of Gal*f*β1 → 2Gal*f*β → 4GlcNAcα → motif appears to be unique in Tc-mucins. Mammalian cells do not produce glycoproteins or glycolipids containing Gal*f*, and this epitope elicits a strong immune response in *T*. *cruzi* infected mice [32].

The *O*-glycans containing both β-Gal*f* and β-Gal*p* linked to → 4GlcNAcα → and → 6GlcNAcα→, respectively, have terminal β-Gal*p* residues available for sialylation. Along this line, the Tc-mucins from Dm28c [28] and Tulahuen [27] strains express *O*-glycan containing as nonreducing end, both sialic acid and Ga*lf* residues (Fig. 1). Using synthetic Gal*f* and Gal*p*-contaning oligosaccharides, with a recombinant preparation of *T*. *cruzi trans*-sialidase, and sialyllactose as sialic donor, Agustí and co-authors [33] verified that the presence of Gal*f* in the O-glycans from Tc-mucins does not impair their acceptor properties. Furthermore, this third biosynthetic pathway forms a trigalactosylated (Fig. 1, G-, Colombiana, Dm28c, Tulahuen strains) glycan, which differs from glycan in Family 1 in that the additional Gal*p* residue is linked β1 → 3 rather than β1 → 2; also, two tetragalactosylated members, which the most common arises by addition of a Gal*p*β1 → 2 to the 3-substituted Gal*p* residue attached to the GlcNAc *O*-6. The presence of a branched tri-galactose structure on the 6-arm appears to be restricted to Core 2. A minor tetragalactosylated glycan (Fig. 1, Tulahuen strain) arises by addition of a β-Gal*f* unit to the Gal*f O*-2. Two pentagalactosylated structures have been also observed, arising by addition of either a β-Gal*p* or a β-Gal*f* residue to *O*-2 of the Gal*f* on the GlcNAc *O*-4, resulting in structures described in Fig. 1 (Tulahuen strains). These data imply the presence of different β-galactopyranosyl transferases expressed in a strain-dependent manner, emphasizing that the Tc-mucin *O*-glycan structures are strain-dependent.

Sialylation of the *T*. *cruzi O*-linked glycans occurs through the action of a parasite-specific *trans*-sialidase [11], which transfers sialic acid from Neu5Acα2 → 3Gal*p*β-containing exogenous donor molecules to terminal βGal*p*-containing acceptors, attaching it in an α2–3 linkage configuration. A mixture of anionic oligosaccharides was isolated from the Tc-mucins of CL-Brener strain [29], which were characterized as 3′-sialyl lactosaminitol, Neu5Acα2 → 3Gal*p*β1-4GlcNAcα1- and two 3′-monosialylated variants, Gal*p*β1 → 4[Gal*p*β1 → 6]GlcNAc, in approximately, equal amounts, suggesting *T*. *cruzi trans*-sialidase has no specificity for the 4- or 6- arm. Although all terminal βGal*p* residues are potential acceptors for sialic acid, no sialylated forms of the more complex Core 2 glycans have been observed, and so any selectivity in the sialylation of the various nonreducing end β-Gal*p* residues remains undefined. Also, no evidence was found for disialylated *O*-glycans. Consistent with data from *in vitro* sialylation of *O*-linked glycans purified from Tc-mucins of epimastigotes [7] and metacyclic trypomastigotes [34]. The incorporation of one molecule of sialic acid hinders entry of a second molecule when two potential acceptor sites are present. The *T*. *cruzi trans*-sialidase substrate donor specificity has been the subject of research for many groups [35, 36], likewise there are different patented processes related to the enzymatic synthesis of sialylα2 → 3βgalactosides, using this enzyme.

## *T*. *cruzi trans*-sialidase (Tc-TS) activity and Tc-mucins

Four points related with the pathogenesis of Chagas disease are importants: (i) Tc-mucins are the main acceptors of sialic acid in *trans*-sialidase mediated reaction [7, 8, 37]; (ii) sialylation of Tc-mucin *O*-glycans is crucial for the viability and persistence of *T*. *cruzi* in mammalian hosts [38–40]; (iii) the initial incorporation of GlcNAc through pp-α-GlcNAcT is a limiting step for the addition of sialic acid by *T*. *cruzi trans*-sialidase (Tc-TS); and (iv) no similar mammalian enzymes were described.

Hundreds of genes encoding Tc-TS enzymes and Tc-mucin glycoproteins are present in the *T*. *cruzi* genome, and, interestingly, Tc-mucins glycoproteins genes are closely linked to members of the *trans*-sialidase super-family at multiple sites in the *T*. *cruzi* genome [41]. The co-expression of TcTS and pp-α-GlcNAcT has been also observed [26]. Furthermore, there are evidences that the increase or decrease of Tc-TS and pp-α-GlcNAcT expressions are dependent upon the different forms of the parasite, during the infectious process [26].

The *T*. *cruzi* TS is an enzyme located on the external surface of the parasite, and a modified sialidase that, instead of releasing sialic acid, can transfer the host-derived sialic acid to terminal βGal*p* in the Tc-mucin *O*-glycans. This enzymatic reaction is different from the known sialyltransferases present in the Golgi that exclusively use CMP-sialic acid as the donor substrate.

The first evidence on a novel pathway for the incorporation of sialic acid into *T*. *cruzi* glycoproteins, through an unusual transglycosylase activity, was done by Previato and co-authors [11]. The authors have observed that *T*. *cruzi* cells grown in the presence of fetal calf serum (sialic acid donor) were agglutinated by wheat germ agglutinin (WGA), a lectin that also recognizes terminal sialic acid units. Nonetheless, in the absence of fetal calf serum in the medium culture, or if the parasites were treated with *Clostridium perfringes* neuraminidase, the WGA binding was abolished and instead the *T*. *cruzi* cells agglutinated by peanut agglutinin (PNA), a lectin that recognizes terminal residues of βGal*p*. Further, these later cells regained their WGA agglutinability when incubated with fetuin or sialyllactose, but not with free sialic acid. These same results were obtained with energy-rich and energy-depleted *T*. *cruzi* cells [11]. Later, the presence of *trans*-sialidase activity was proven, and established that the expression of TcTS and the acquisition of sialic acid by *T*. *cruzi* are relevant events in the interaction and invasion of the parasite to the host [38, 42].

## Developmental life cycle of *T*. *cruzi* and Tc mucin functions

*T*. *cruzi* presents a complex life cycle involving the hematophagous triatomine insect and mammalian host species, with different developmental stages. Within the insect, *T*. *cruzi* differentiates in two diverse forms: replicative epimastigote and non-replicative metacyclic trypomastigote forms. Metacyclic trypomastigotes are mostly transmitted during a blood meal of the insect, which are able to invade a wide variety of mammalian nucleated cells. In mammalian hosts, *T*. *cruzi* behaves as an obligate intracellular pathogen. Inside the cell, the infective trypomastigotes are temporarily contained in the parasitophorous vacuole subsequently the parasites escape to the cytosol, and differentiate into the replicative amastigotes, which after several divisions, transform into cell-derived trypomastigotes, which are released into the bloodstream. The *T*. *cruzi* life cycle closes when a triatomine vector feeds on a *T*. *cruzi*-infected mammal [43]. Several mechanisms of infection have been proposed for the extremely complex *T*. *cruzi*-host cell interaction process, which involves many putative *T*. *cruzi* ligands and a growing list of host cell targets [44, 45].

Here, we summarize the main aspects of *T*. *cruzi*-host cells interactions, involving Tc-mucins. Tc-mucins from epimastigotes and metacyclic trypomastigotes differ from those of cell culture trypomastigotes in their apparent molecular masses. The Tc-mucins isolated from *T*. *cruzi* insect forms migrate on SDS-PAGE as a broad band in 35–50 kDa range [8, 11], while Tc-mucins from cell-derived trypomastigotes present a wide range from 60 to 200 kDa molecular masses [30, 42], these are equivalent to the highly glycosylated protein sharing sialic acid-containing epitopes crucial for mammalian cell attachment and invasion [42]. Despite the relevance of cell-derived trypomastigotes, little is known about the chemical structure of *O*-glycans of Tc-mucins from these forms, however a key difference from the insect-stage mucins, is the presence of terminal α-galactopyranosyl residues, which are targets of lytic antibodies isolated from patients with chronic Chagas disease, in cell-derived trypomastigote mucins [30, 31]. Tc-mucins from cell-derived trypomastigotes induce the synthesis of nitric acid and proinflammatory cytokines IL-12 and TNF-α by stimulated macrophages [46], effects that may be modulate the immune response to *T*. *cruzi* during the infection. Many of the biological properties of Tc-mucins have been related to the presence of sialic acid-containing α-*O*-linked glycans [42]. It has been demonstrated that Tc-mucins are the main acceptors of sialic acid in *trans*-sialidase mediated reaction, and that the sialylation of Tc-mucin O-glycans is crucial for the viability and persistence of these parasites. Nevertheless, Yoshida and co-author [47] demonstrated that the ability of cell-derived trypomastigotes, obtained from *T*. *cruzi* G-strain, to invade HeLa cells is independent of sialic acid, providing evidence that the rate of invasion of desialylated parasites is significantly higher after treatment of both *T*. *cruzi* and purified Tc-mucin with neuraminidase. However, this effect seems to be strain-dependent. The major structural features do not differ between *O*-glycans from epimastigote [7] and metacyclic trypomastigote forms [34], however, mucins from metacyclic trypomastigotes, but not from epimastigotes, bind to cultured cell lines [48]. The involvement of Tc-mucins from metacyclic trypomastigotes, in invasion to mammalian cell lines, was verified by inhibition of parasite internalization by monoclonal antibodies that recognize Gal*p* or Gal*f*-containing epitopes of *O*-glycans [49]. The mechanism of interaction of metacyclic trypomastigotes-host cells mainly relies on *T*. *cruzi* strains which express on their surface variant forms of *O*-glycan and exhibit diverse range of capability to invade host cells *in vitro* [50, 51].

## Conclusion

The surface of *T*. *cruzi* is covered by mucin-like molecules (Tc-mucins) which are implicated to parasite protection in both vertebrate and invertebrate hosts, in mechanisms of infectivity and modulation of the host immune response throughout the *T*. *cruzi* life cycle. The obvious medical significance of *T*. *cruzi* and the knowledge of the molecular structure of the Tc-mucin have led to intensive study of its biosynthesis. The first step of *O*-glycosylation of Tc-mucins is a unique biosynthetic pathway catalyzed by a pp-α-GlcNAcT, which transfers α-*O*-*N*-acetylglucosamine (GlcNAc) to threonine (Thr) residues.

The pathways leading to *O*-glycosylation of *T*. *cruzi* glycoproteins show unusual features when compared to that of mammalian cells. Optimistically, the selective expression of enzymes, which are not present in the parasite’s hosts, such as the pp-α-GlcNAcT and TcTS, an enzyme with a unique specificity for the addition of sialic acid on Tc-mucins, might provide suitable novel targets for the development of less toxic and more effective treatments against Chagas’ disease.
